# The Human Behaviour-Change Project: harnessing the power of artificial intelligence and machine learning for evidence synthesis and interpretation

**DOI:** 10.1186/s13012-017-0641-5

**Published:** 2017-10-18

**Authors:** Susan Michie, James Thomas, Marie Johnston, Pol Mac Aonghusa, John Shawe-Taylor, Michael P. Kelly, Léa A. Deleris, Ailbhe N. Finnerty, Marta M. Marques, Emma Norris, Alison O’Mara-Eves, Robert West

**Affiliations:** 1 UCL Centre for Behaviour Change, University College London, 1-19 Torrington Place, London, WC1E 7HB UK; 2 EPPI-Centre, Department of Social Science, University College London, London, UK; 3 Health Psychology, University of Aberdeen, Scotland, UK; 4 IBM Research – Ireland, Dublin, Ireland; 5 Department of Computer Science, UCL, London, UK; 6 Primary Care Unit, Institute of Public Health, University of Cambridge, Cambridge, UK; 7 Department of Epidemiology and Public Health, University College London, London, UK

**Keywords:** Behaviour change interventions, Implementation, Ontology, Machine learning, Natural language processing, Evidence synthesis, Artificial intelligence

## Abstract

**Background:**

Behaviour change is key to addressing both the challenges facing human health and wellbeing and to promoting the uptake of research findings in health policy and practice. We need to make better use of the vast amount of accumulating evidence from behaviour change intervention (BCI) evaluations and promote the uptake of that evidence into a wide range of contexts. The scale and complexity of the task of synthesising and interpreting this evidence, and increasing evidence timeliness and accessibility, will require increased computer support.

The Human Behaviour-Change Project (HBCP) will use Artificial Intelligence and Machine Learning to (i) develop and evaluate a ‘Knowledge System’ that automatically extracts, synthesises and interprets findings from BCI evaluation reports to generate new insights about behaviour change and improve prediction of intervention effectiveness and (ii) allow users, such as practitioners, policy makers and researchers, to easily and efficiently query the system to get answers to variants of the question ‘*What works, compared with what, how well, with what exposure, with what behaviours (for how long), for whom, in what settings and why?’*.

**Methods:**

The HBCP will: a) *develop an ontology of BCI evaluations and their reports* linking effect sizes for given target behaviours with intervention content and delivery and mechanisms of action, as moderated by exposure, populations and settings; b) *develop and train an automated feature extraction system* to annotate BCI evaluation reports using this ontology; c) *develop and train machine learning and reasoning algorithms* to use the annotated BCI evaluation reports to predict effect sizes for particular combinations of behaviours, interventions, populations and settings; d) *build user and machine interfaces* for interrogating and updating the knowledge base; and e) *evaluate all the above* in terms of performance and utility.

**Discussion:**

The HBCP aims to revolutionise our ability to synthesise, interpret and deliver evidence on behaviour change interventions that is up-to-date and tailored to user need and context. This will enhance the usefulness, and support the implementation of, that evidence.

**Electronic supplementary material:**

The online version of this article (10.1186/s13012-017-0641-5) contains supplementary material, which is available to authorized users.

## Background

Many global threats to human health and wellbeing can only be solved by people, organisations and governments changing their behaviour. This includes behaviours directly relevant to health but also behaviours of policy-makers and providers responsible for promoting health and delivering healthcare. To that end, we need to use evidence being gathered about behaviour change more effectively than at present. A great deal more evidence is produced and published than it is possible for researchers to be able to use effectively with conventional methods.

The current waste in research is being increasingly recognised and addressed: for example, the Lancet series “Research: increasing value, reducing waste” [1] and the subsequent REWARD (REduce research Waste And Reward Diligence) campaign [2]. The waste occurs in biomedical and behavioural sciences and is apparent at every stage of the research process, including poor reporting of research so that evidence cannot be synthesised and implemented effectively and efficiently. The potential for implementation science to improve health promotion and delivery will remain compromised unless the problem of this waste is tackled.

The quantity, complexity and variability of reporting of behaviour change intervention (BCI)^g^ evaluations (see Table 1 for glossary of definitions for terms identified with the superscript ^g^) severely limit the accessibility and value of this evidence for those who need it (Optimising the value of the evidence generated in Implementation Science: the use of ontologies to address the challenges, Invited submission forthcoming). The Human Behaviour-Change Project (HBCP) will develop and evaluate a *BCI Knowledge System* ^*g*^: an automated system delivering comprehensive, high quality, timely and accessible syntheses and interpretations of evidence.

**Table 1:** Glossary of terms

Term	Definition	Source
Algorithm	Sequence of actions to perform calculation, data processing and automated reasoning tasks.	
Annotation	Process of identifying selections of content from BCI evaluation reports describing features of BCI evaluations, together with specification of the features described using the BCIO.	
Artificial Intelligence (AI)	The theory and practice of building computer programs to perform tasks that a human would reasonably regard as requiring intelligence.	[31]
Attribute	A quality or disposition of an object, collection of objects, process or collection of processes.	[10]
Automated annotation	Annotation that is undertaken by a computer program.	
Basic Formal Ontology	An upper level ontology consisting of continuants and occurrents developed to support integration especially of data obtained through scientific research.	[10]
BCI database	The database containing all information about BCI evaluation reports and inferences from these, organised according to the BCIO.	
BCI evaluation	A comparison between two or more BCI scenarios focusing particularly on estimating the differences in outcomes between these scenarios.	
BCI evaluation report	Description of a BCI evaluation, usually in the form of a published research report.	
BCI Knowledge System	An automated system delivering comprehensive, high quality, timely and accessible syntheses and interpretations of evidence in the domain of behaviour change.	
BCI ontology (BCIO)	An ontology that represents entities and relationships related to BCI evaluations and their reports.	
BCI scenario	A scenario (a sequence or development of events) consisting of a BCI, its target behaviours, and factors that influence the outcome of the BCI in relation to the target behaviour.	
Behaviour	Anything a person does in response to internal or external events. Actions may be overt (motor or verbal) and directly measurable or, covert (activities not viewable but involving voluntary muscles) and indirectly measurable; behaviours are physical events that occur in the body and are controlled by the brain.	[32]
Behaviour Change Intervention (BCI)	A product, service, activity or structural change, intended to achieve behaviour change. It can be specified in terms of the content of the intervention and the way this is delivered.	[33]
Behaviour Change Technique (BCT)	The smallest component of an intervention compatible with retaining the postulated active ingredients, and can be used alone or in combination with other BCTs.	[34]
Behaviour Change Techniques Taxonomy version 1 (BCTTv1)	A hierarchical classification system (taxonomy) for reliably specifying intervention components in terms of 93 well-defined behaviour change techniques (BCTs), organised into 16 groupings.	[35]
Cochrane Collaboration	A global independent network of researchers, professionals, patients, carers and people interested in health. It is a not-for-profit organisation with contributors from more than 120 countries working together to produce credible, accessible health information that is free from commercial sponsorship and other conflicts of interest. They work to produce reviews that summarise the best available evidence generated through research to inform decisions about health.	[36]
Context	Features of a BCI scenario, independent of the BCI itself that may influence the outcome.	[33]
Delivery	Features of a BCI related to the manner in which the intervention is enacted.	[33]
Effect	The estimated effect size for the combination of intervention, usage (exposure and engagement), context, mechanism of action and behaviour, always specified in relation to a comparator.	[33]
Engagement	The amount and manner of use of, or interaction with, an intervention among people who use it at least to some degree.	[33]
Entity	Anything that exists, that can be a continuant or an occurrent as defined in the BFO.	
Exposure	Factors relating to the interaction between the intervention and the target population (the extent and nature of the target population’s access to and engagement with the intervention) that may influence the intervention’s effect. Consists of reach and engagement.	
Extensible Markup Language (XML)	A markup language that defines a set of rules for encoding documents in a format that is both human-readable and machine-readable.	
Feature	An instance of a BCIO entity that forms part of a BCI evaluation or BCI evaluation report.	
Human Computer Interaction (HCI)	An area of study that focuses on ways in which humans and computers interact.	
Knowledge base	A repository of information from the domain of interest linking classes into the ontology to instances.	[11]
Machine learning (ML)	Computer algorithms that learn from sample inputs and apply that learning to make predictions on data or classify data into categories.	[37]
Mechanism of Action	Process that mediate the effect of the intervention on the behavioural outcome. These can be specified in terms of changes to capability, opportunity, motivation or other behaviours.	[28]
Method	The set of features of methods used in BCI evaluations, containing features relating to study design (e.g., controlled trial), measures, sample identification and recruitment, sample size, and risk of bias.	
Natural Language Processing (NLP)	Algorithms that extract meaning from passages of text in a form that can be used for inference by computers.	[38]
Object	A material entity that is 1) spatially extended in three dimensions, 2) causally unified, and 3) maximally self connected.	[10]
Ontology	A standardised representational framework providing a set of terms for the consistent description (or “annotation” or “tagging”) of data and information across disciplinary and research community boundaries.	[10]
Outcome	Absolute numerical value of target behaviour associated with a BCI scenario.	
OWL	A formal language for describing ontologies. It provides methods to model classes of “things”, how they relate to each other and the properties they have. OWL is designed to be interpreted by computer programs and is extensively used in the Semantic Web where rich knowledge about web documents and the relationships between them are represented using OWL syntax. In the HBC project elements of OWL are used to express ontologies relevant to behaviour change in a way that can be processed by reasoning and machine learning systems.	[39]
PICO	An ontology used by Cochrane that represents important entities in medical and population science, focusing on evaluations of clinical and public health inteventions. The acronym stands for: **P**atient, **P**opulation or **P**roblem, **I**ntervention, **C**omparison (group intervention is compared to) and **O**utcome.	[40]
Population	Characteristics of the individuals, groups, sub-populations or populations whose behaviour one is seeking to change, including their other behaviours, mental health status etc.	[33]
Process	An entity that exists in time by occurring or happening, has temporal parts, and always depends on at least one object as participant.	[10]
Reach	Uptake of intervention.	[33]
Reasoning algorithms	Computer programs that can generate conclusions from available knowledge. In the HBCP project, reasoning algorithms may derive conclusions through combinations of logic based reasoning (where basic axioms about the behaviour of the environment are provided as a basis for reasoning) and statistical learning (where patterns are used to construct new facts).	
Risk of bias feature	Features of BCI evaluation method and reporting that may lead to the reported effect size of the evaluation not being accurate.	[41]
Setting	Features of the social and physical environment that may influence the outcome of a BCI.	[33]
Target Behaviour	Behaviour that a BCI seeks to influence.	
Taxonomy	A classification system in which classes are uniquely assigned to a higher level class.	[42]
User Interface (HBCP)	The means by which the user and a computer system interact, in particular the use of input devices (keyboards, screens etc) and software. The HBCP interface will consist of a machine interface and a human user interface. The machine interface will provide application programming interfaces that will allow other programs to query and provide information to the BCI Knowledge System (e.g. results of searches for BCI evaluation reports). The human user interface will be a website and associated supporting programs to allow users to query and inform the BCI Knowledge System.	[43]

### The challenges of a rapidly expanding, complex evidence base

BCIs^g^ are policies, activities, services or products designed to induce or support people to act differently from how they would have acted otherwise. They involve attempting to change either characteristics of members of the target population (in terms of their knowledge, skills, beliefs, feelings or habits), or their social or physical environment, or both. In the large majority of cases, the goal is to achieve change that is sustained over an extended period of time (e.g., reducing excessive alcohol consumption or smoking prevalence in the general population, or fostering new prescribing patterns among clinicians). Research findings have the potential to provide invaluable knowledge to help with developing or selecting BCIs but this evidence needs to be synthesised and interpreted. We need a cumulative, contemporaneous and accessible knowledge base^g^ of behaviour change findings to continue to build the science of human behaviour change.

Systematic reviews and meta-analyses provide a means of gathering and synthesising this evidence but the scientific literature on behaviour change is vast and accumulating exponentially. Considering the person-hours required for any given review, there are neither the human nor financial resources to achieve this manually at the scale required. Insufficient human resources to undertake evidence reviews and syntheses also means that these are often out of date by the time of completion [3]. The median time for primary study results to be incorporated into a systematic review has been found to range from 2.5 to 6.5 years [4] and only a minority of reviews are updated within 2 years of publication [5]. A further limitation of the current method is that there is often insufficient power in the evidence gathered to enable moderator analyses, especially for under-researched populations and geographical areas.

In addition, the diversity in the literature presents considerable challenges when it comes to making generalisations in terms of intervention effectiveness. Target behaviours^g^ vary widely in their characteristics, from cessation of unwanted behaviours such as tobacco smoking to increases in desired ones such as implementing evidence-based practice. The types of interventions evaluated are also subject to wide variation from policies such as raising excise duty on unhealthy products to digital mobile applications for promoting medication adherence. Populations^g^ also vary, with some studies involving what are intended to be general population samples and others based on participants with special characteristics, such as mental health problems. Settings^g^ vary across dimensions from physical locality to culture. With such diversity in the evidence base, there is a need for a coherent conceptual framework to allow evidence from different studies to be integrated and compared.

Addressing heterogeneity in the research literature is made more challenging by inconsistent and incomplete reporting of interventions and study methods and findings. The situation has been improved by the publication of a number of guidelines [6], but intervention evaluations still vary widely in quality and format, and are reported inconsistently and incompletely using terminology with limited standardisation [7].

Methods of evidence synthesis such as meta-analysis and meta-regression have substantially improved the ability to draw generalisable conclusions from intervention evaluations, but they are mostly limited to making inferences about simple effects for interventions that have been evaluated, or first-order interactions with moderator variables. More advanced statistical techniques are beginning to be developed [8], and will need to be built on. There is a need to be able to draw inferences that take account of complex interactions between intervention characteristics, populations and settings. Moreover, even with the numbers of studies retrievable by current methods, the populations and settings to which one may wish to generalise are so varied that making inferences from studies to real-world applications is problematic.

Important challenges facing evidence synthesis and interpretation, and approaches to addressing those challenges are shown in Table 2.

**Table 2:** Challenges facing evidence synthesis and interpretation in behaviour change

Challenge	Solution
*Research methods:* Diversity of research methods and topics, and inconsistency and incompleteness in reporting of study methods and findings	Development and application of an ontology of behaviour change interventions
*Human limitations:* Insufficient human resources to undertake reviews and syntheses in a timely manner given the volume of findings and increasing rate of evidence accumulation	Use of automated literature searching and study feature extraction
*Research findings:* Equivocal or contradictory findings, sparseness of findings relative to the number and variety of behaviours, interventions, populations and settings about which information is required, complexity of interactions between intervention components, populations, settings and behavioural outcomes	Use of machine learning and reasoning algorithms for evidence synthesis and interpretation. Focus will be on methods providing a confidence level associated with the prediction so as to be able to rigorously incorporate conflicting and missing information

### The Human Behaviour-Change Project (HBCP)

The vision for the Human Behaviour-Change Project [9] is to build a Knowledge System that accesses the growing number of BCI evaluation reports^g^, automatically annotates these reports to identify key features^g^, and synthesises and interprets the findings to answer variants of the big question: *‘What works, compared with what, how well, with what exposure, with what behaviours (for how long), for whom, in what settings and why?’.* The project includes the development of a user interface^g^ to allow intervention designers, policymakers, researchers, the general public and other computer systems to access, interrogate and update the knowledge base.

A multi-disciplinary team, spanning behavioural, computer and information scientists and system architects, supported by substantial engagement from scientists and users, will develop and evaluate the first iteration of the HBCP establishing proof of principle, with an initial focus on smoking cessation. This domain was selected due to its large and relatively well-defined evidence base and outcome measures that are relatively robust and important for public health.

### Organising and classifying research, and generating inferences: The role of ontologies

The process of knowledge accumulation requires a common conceptual framework within which information can be represented. Data structures that organise knowledge in a structure that specifies entities^g^ and their relationships are called ‘ontologies’^g^ [10, 11].

In information science an ‘ontology’ is defined as a data structure consisting of a set of 1) unique identifiers representing types of ‘entity’^g^ (primarily objects^g^, attributes^g^, processes^g^, or collections of these), 2) labels and definitions corresponding to these identifiers, and 3) specified relationships between the entities. The labels and definitions of entities and relationships in a given ontology^g^ make up a ‘controlled vocabulary’ which provides a basis for the interoperability of databases using the ontology [10, 11].

Ontologies have transformed a number of areas of science. Most notably the Gene Ontology has unified the field of biology which previously was highly fragmented [12]. Ontology development requires considerable expertise and to that end the OBO Foundry [13] was established to provide a resource for ontology developers and a set of guiding principles from which to work.

As yet, no widely-used ontology has been developed for behavioural science, although ones have been developed for public health [14] and mental entities such as emotions [15], mental disorders and mental functioning [16]. An ontology for understanding human behaviour change needs to represent both causal relationships (e.g., that a given type of intervention affects a given behaviour in a specified context) as well as semantic relationships (e.g., that a given type of intervention is a subclass of a broader type of intervention) [10, 11].

**Fig. 1:**
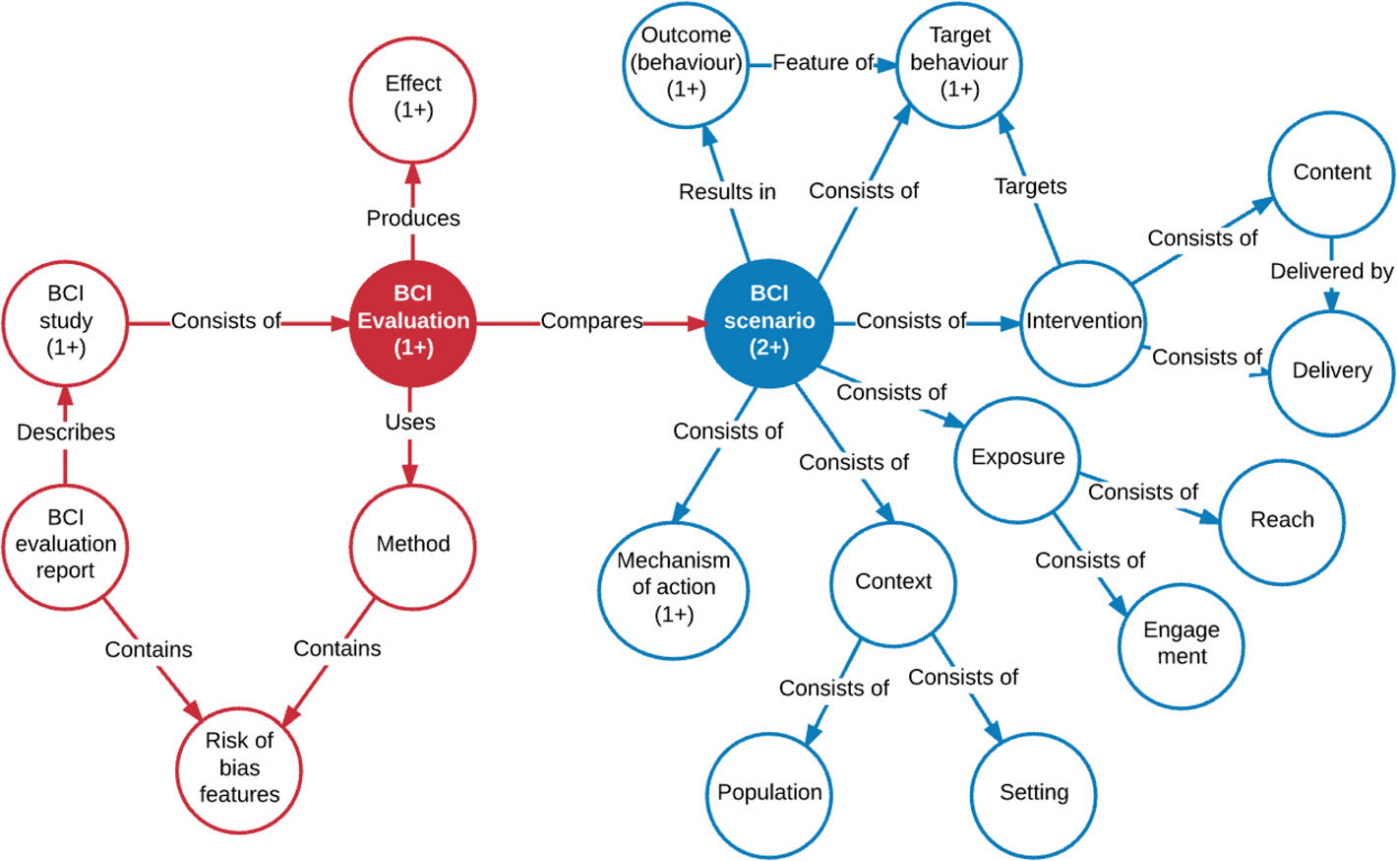
Key upper-level entities and examples of relationships to be captured in the BCIO. Numbers in brackets refer to the number of entities required if not 1

The HBCP will develop a BCI ontology (BCIO^g^) that will define important entities described in BCI evaluation reports. Fig. 1 shows upper-level entities that need to be captured in the BCIO and some of their relationships. The labels for these may change in the course of development of the BCIO but this provides an indication of what information needs to be captured. Note that Fig. 1 is not the formal ontology but is shown to illustrate key parts that need to be included.

**Fig. 2:**
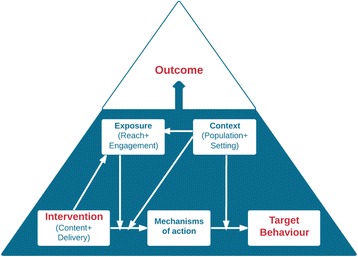
Upper-level entities in BCI scenarios, and their causal connections

The BCIO includes entities that are important in answering questions about BCI effectiveness as follows:

- **BCI evaluation report** is a written description of a BCI study, which provides information about one or more BCI evaluations (see below), including the intervention(s) being evaluated, study methods and findings. It will typically involve a published paper but may include information from more than one paper, for example if important features of the methods are described in a protocol paper.
- **BCI study** is an empirical data-gathering activity consisting of one or more BCI evaluations.
- **BCI evaluation** is a comparison between two or more BCI scenarios^g^.
- **Method** ^**g**^ defined as the set of attributes of BCI evaluation methods. These include study design (e.g., controlled trial), measures, sample identification and recruitment, sample size, and ‘quality’
- **Effect** ^**g**^ defined as the result of a comparison between outcomes of each pair of intervention and comparator scenarios. It is specified in terms of an effect descriptor (e.g., odds ratio, risk difference), effect size and confidence intervals.
- **Risk of bias features** ^**g**^ are features of the BCI evaluation report and method that may have an impact on the observed effect of a BCI evaluation. These include study design, blinding, method of randomisation etc.
- **BCI scenario** ^**g**^ is a scenario (a sequence or development of events) consisting of a BCI, its target behaviours, and factors that influence the outcome of the BCI in relation to the target behaviour (Fig. 2). A BCI scenario may be hypothetical (if it is one that is being considered for modelling purposes), planned (if it is one that is or has been intended), or realised (if it has been enacted, for example in a BCI evaluation). When annotating BCI evaluation reports (see below) the aim is to capture the realised BCI scenarios based on information from the reports. When querying the knowledge base (see below) the aim will be to present features of a planned or hypothetical BCI scenario with a view to obtaining a prediction of the likely outcome.
- **Outcome (behaviour)** ^**g**^ defined as the type(s) of behaviour that the BCI seeks to change (e.g., tobacco smoking) together with a collection of attributes (e.g., duration, frequency or incidence) that together make specific types of outcome measure (e.g., self-report of not smoking for 6 months supported by a salivary cotinine concentration of less than 15 ng/ml measured at the final follow up point) [17].
- **Intervention** ^**g**^ defined as a set of types of policies, activities, services or products that are intended to result in a specified outcome in relation to the target behaviour. The intervention is specified in terms of summary descriptors (e.g., ‘brief opportunistic advice from a GP on smoking’) together with detailed descriptions of ‘content’^g^ such as the techniques used (e.g., pharmacological support, verbal persuasion about capability etc.), and ‘delivery’^g^ (e.g., 5 min, single session, verbal, face-to-face, during a routine consultation, by GP, trained with UK National Centre for Smoking Cessation Very Brief Advice online course). The term ‘intervention’ is also used to refer to any comparator in a BCI evaluation (e.g., usual care).
- **Context** ^**g**^ defined as factors (consisting of characteristics of the population and setting) not directly connected with the intervention that may influence the intervention’s effect.
- **Exposure** ^**g**^ defined as factors relating to the interaction between the intervention and the target population (the extent and nature of the target population’s access to and engagement with the intervention) that may influence the intervention’s effect. Consists of reach^g^ (e.g., the proportion of the target population that has access to, or is exposed to, the intervention) and engagement^g^ (e.g., the extent and nature of the target population’s interaction with intervention components).
- **Mechanism of action** ^**g**^ defined as the type(s) of process by which interventions influence the target behaviour (e.g., through increasing strength and frequency of feelings of concern about the risks of an unhealthy behaviour; providing a physical or social cue to action).
- **Outcome (behaviour) value** ^**g**^ defined as the value attaching to the target behaviour for a given BCI scenario (e.g., the outcome would be 15% of the population where the target behaviour was six months of continuous abstinence from smoking).

The entities in the BCI scenario interact in specific ways, as showed by the arrows in Fig. 2. The content and delivery of an intervention influences the target behaviour through one or more mechanisms of action. The context moderates the influence of 1) the intervention on the mechanism of action and 2) the mechanism of action on the behaviour. Exposure moderates the influence of the intervention on the mechanism of action and is itself influenced by the intervention and context.

Thus if a GP prescribes nicotine replacement therapy (intervention) to smokers interested in stopping (population), as part of a routine consultation in a GP surgery in the UK (context), and 60% of smokers obtain the medication and start the treatment, and 50% take it as prescribed (exposure), this may reduce cigarette cravings (mechanism of action) and so lead to at least 6 months of abstinence (outcome behaviour) in 15% (outcome value) of cases [18].

If one were to conduct a study to assess the effect of GPs prescribing nicotine replacement therapy, this scenario would be compared with a BCI scenario such as GP advice without the offer of a prescription. The comparison would have a number of features relating to study design (e.g., RCT), sample recruitment and selection, sample size, baseline and outcome measures etc. The comparison of outcomes between the two scenarios would constitute the ‘effect’ of the prescription intervention relative to advice without a prescription, expressed in terms of an odds ratio or risk ratio with a corresponding confidence interval. The observed effect would therefore be a function of the features of the intervention and comparator BCI scenarios together with the study methods (Fig. 1).

### The role of computer science in the HBCP

Artificial intelligence (AI^g^) and machine learning (ML^g^) applications have been developed to generate and interrogate large, accumulating knowledge bases using ontological approaches. In the HBCP, building computer programs to extract and process knowledge from text documents at a level that is usable by experts in the domain, requires several elements that can generally be equated with *intelligence*, such as advanced reading ability and significant domain understanding. In this respect, a computer program performing this task can be thought of as artificially intelligent.

Building computer programs to perform tasks such as recognising patterns in text is usually achieved by applying a technique called *statistical learning,* where a computer program uses example patterns and examples from a training set to construct a statistical model of how a task should be performed. This model can then be generalised to process new, unseen data thereby performing the desired task with high confidence. The technique is statistical because the computer program uses weightings learned from statistical properties of the training examples - for example - frequencies with which important words appear in text.

Other approaches to artificial intelligence, such as logic-based reasoning have been successful in domains such as robotics and sensor-based systems. Here axioms or rules describe the behaviour of the world allowing a computer program to decide how to respond to inputs. Since the HBCP is concerned with learning patterns from text it is expected that statistical learning, rather than other approaches such as logic-based learning, will be most appropriate.

Artificial intelligence and machine learning have been used successfully, for example, in banking customer service [19], and in areas of medicine [20–22]. IBM’s ‘Watson Oncology’ uses AI and ML to extract information from research publications to help clinicians identify appropriate treatment options. Algorithms^g^ are used for entity recognition, information extraction, semantic query expansion in information retrieval, pattern detection, sentiment analysis, and reasoning [23–26].

In the HBCP, computer scientists will develop automated processes to annotate BCI evaluation reports in terms of key features defined according to the BCIO. These will populate a database^g^ structured according to the BCIO. Automated annotation^g^ will require developing and training ‘natural language processing’ (NLP^g^) algorithms and other systems for extracting features from tables and graphs. ML together with reasoning algorithms^g^ will then be used to synthesise and interpret the findings to answer questions and make predictions about what would be expected in as yet unstudied scenarios^g^.

Evidence from studies of human-computer interaction^g^ (HCI) will inform the development of the user interface through which people will use the system. Different groups of users will have different requirements and concerns, which will be addressed in the way that information is presented, and the functionalities available for interacting with it. Understanding user interaction in this project is particularly important, given the ‘black box’ nature of the knowledge base that people will be querying. Addressing concerns relating to the Knowledge System’s trustworthiness, and how the reliability of its predictions can be evidenced, are likely to be particularly important.

## Aim and research questions

**Fig. 3:**
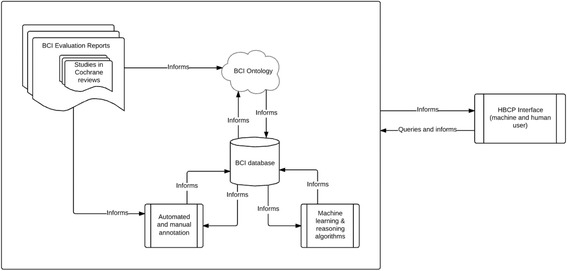
Components of the BCI Knowledge System in the Human Behaviour Change Project

The aim is to develop and evaluate the first generation of a BCI Knowledge System consisting of: the first version of the BCIO; a continually growing database of annotated BCI evaluation reports and inferences drawn from these; algorithms used to create the annotations and draw inferences; and an interface that will allow human users and other computer systems to query and update the database of annotations and inferences. Fig. 3 shows the main components of the BCI Knowledge System that is proposed and how they interact.

The main research questions fall into two categories: (1) those relating to creation of the BCI Knowledge System (the BCIO, the database of annotated BCI evaluation reports, the automated feature extraction algorithms used to annotate these reports, the ML and reasoning algorithms used to synthesise the evidence and draw inferences, stored inferences, and the interface), and (2) those relating to evaluation of the BCI Knowledge System.

### 1. Creating the BCI knowledge system

i. What are the key features that need to be captured from BCI evaluation reports and models of behaviour change to build the BCIO? In particular, how should we represent: i) the content and delivery of interventions and comparators; ii) exposure to interventions and comparators in terms of reach (whether the intervention/comparator reached the sample studied) and how far and in what ways the targeted population engaged with the intervention and comparator; iii) targeted behaviours in terms of type of behaviour, duration and specific outcome measures; iv) contexts in terms of the target populations and settings; v) putative mechanisms of action of the intervention, vi) outcomes and effects in terms of the statistical estimate used (e.g. rate ratio) and confidence intervals, vii) study methods and reporting features, including those that influence the weight that should be given to the evaluation and the risk of bias.
ii. What automated feature extraction algorithms (i.e., combinations and extensions of NLP components) can be developed and trained to extract relevant information from BCI evaluation reports in order to create the database of annotated reports?
iii. What ML and reasoning algorithms can be developed to synthesise evidence using the database of annotated reports and the BCIO to arrive at i) inferences regarding BCI effectiveness and ii) confidence estimates associated with those inferences?
iv. What are the key features of a user interface that make it easy to use and provide answers that are understood and trusted?

### 2. Evaluating the output

i. What is the inter-rater reliability of the manual annotation system for the BCIO?
ii. What is the accuracy of the automated feature extraction system in annotating BCI evaluation reports?
iii. What is the accuracy of the predictions and associated confidence estimates generated by the ML and reasoning algorithms?
iv. How far does the BCI Knowledge System add value over existing methods of evidence synthesis? For example, can automated reviews produced by the system improve upon systematic reviews conducted by humans (and if so, by how much)?
v. What are users’ assessments of the system’s accuracy, salience, validity, and utility?
vi. What new insights about behaviour change are generated by the system?
vii. How can information be conveyed most effectively and efficiently between the BCI Knowledge System and users of different types (e.g. scientists, expert users, practitioners, policy makers)?

## Methods

### Overview

Six sets of activities will be undertaken, much of the work being conducted in parallel: 1) forming and engaging with stakeholder groups; 2) developing the BCIO; 3) annotating BCI evaluations according to the BCIO using manual and automated processes and building the BCI database^g^; 4) developing and applying ML and reasoning algorithms to draw inferences in response to queries; 5) developing an interface for users and other applications to query the system and provide feedback that can be used to update the BCI Knowledge System as a whole; and 6) evaluating the BCI Knowledge System and its components.

Details of the methodological approach being taken to BCI Ontology development, manual annotation of BCI evaluation reports and the development of automated annotation algorithms, machine learning and reasoning algorithms are presented in Additional file 1. Methods of working will be made accessible in Open Science Framework [27] as they are updated. Outputs and processes of the HBCP will be made available to potential collaborators who are interested in applying these or conducting complementary projects. We will engage a wide variety of stakeholders in a number of groups to enable engagement across countries, cultures, academic disciplines and behavioural domains. A summary of engagement methods are outlined in Additional file 2.

### Development of the HBCP interface

An interface will be developed to facilitate querying and updating the knowledge base, and the BCI Knowledge System as a whole. It will consist of a machine interface and a user interface.

The machine interface will provide the primary means by which BCI reports are added to the database. It will provide a facility by which programs that search and screen reports can feed those that are relevant into the database, ready for annotation. It will also include an application programming interface (API) to allow for other programs to formulate queries and receive responses in machine readable form. The aim is to make the BCI Knowledge System as interoperable as possible with other software that is being, and will be, developed.

The user interface will be a website that will build on the wide range of external perspectives that have fed into the BCIO development and ML components of this work and engagement with a wide range of stakeholders. It will handle three types of scenario:

1. Users will be able to query the system and obtain results in multiple forms (e.g., lists of individual studies, synthesised data, and inferences from the BCI database). The interface will come in several forms that are tailored for particular groups of users.
2. HBCP stakeholders will be able to interact with the BCIO, the BCI database, and the individual BCI reports in a flexible way. For example they will be able to propose scenarios specified using a purpose-built syntax and conduct sensitivity analyses in which particular studies are included or excluded. They will need elevated privileges for some tasks (e.g., direct editing of annotated research reports).
3. Members of the HBCP research team will be able to use the interface to evaluate, develop and refine the BCIO and ML and reasoning algorithms.

Users of the interface will be able to generate queries about BCI scenarios. They will enter fixed or constrained parameters (e.g., the behavioural outcome, the mode of delivery, the target population, the setting, or a range of effect sizes) and interrogate the BCI knowledge base for predicted values of BCIO entities that are left open. Examples of queries are shown in Table 3.

**Table 3:** Examples of queries from different user groups

Type of user	Requirement	Query
Directors of Public Health for a consortium of Local Authorities in a deprived region of England	To identify effective messaging in a mass media campaign to promote smoking cessation in their region	What is the optimal content, timing and patterning of delivery of a mass media campaign that aims to increase attempts to stop smoking in economically deprived smokers in the North East of England?
A research team developing a research proposal to evaluate a mindfulness smartphone application to promote healthier eating	To find out what evidence there is on whether mindfulness interventions can help people to achieve lasting change in eating patterns	What is the knowledge base on the effectiveness of mindfulness interventions in achieving long-term behaviour change, and are there any general conclusions that can be drawn from this about who it works for, for what behaviours and delivered in what ways?
Highways England (responsible for safety on major roads)	To re-evaluate policies on speed cameras as a way of reducing excessive speed	What effect if any have speed cameras had on reducing the incidence of driving above the speed limit on major roads in England? Are there any factors that influence the effect in terms of geography, type of road, and type of road user?
NHS England	To develop a national campaign to reduce hospital acquired infections through improved hand-hygiene	What interventions have been found to be effective in improving hand hygiene in hospitals? How effective are they? Are some more effective than others? Are there contextual factors (population and setting) that influence the effect of these interventions, and if so what are they?
A cancer charity	To identify the most effective strategies for increasing ultra-violet protection behaviours	What is the relative effectiveness of different interventions aimed at increasing the ultra-violet protection behaviours? How far is this influenced by contextual factors (population and setting)?

Because users will vary in their levels of expertise in the topic of the query, the user interface will provide a facility to guide them through the generation of the query so that they arrive at the most useful results. For example, users may start the query at too general a level of abstraction for the Knowledge System to be able to generate meaningful results, or they may not be aware of the importance of particular moderators or intervention components when generating the query. The user interface should be able to draw attention to these issues and prompt users to generate queries that get the most out of the data available.

Users will also be able to use the interface to generate a curated and annotated bibliography of research reports relevant to their query. This may be particularly useful for systematic reviewers who may want to take advantage of the precision with which the system will permit searches to be carried out, but may want to undertake data extraction and synthesis by hand or using a different program.

### Evaluation of the BCI knowledge system

The HBCP involves evaluation of BCI Knowledge System as a whole as well as its parts. There will be an ongoing process of evaluation and development throughout the project, but at a certain point it will be necessary to assess to what extent the project has met its objectives, and to provide information to guide future decisions. In accordance with the HBCP research questions, the HBCP will undertake the following assessment:

i. *The adequacy, applicability, and validity of the BCIO.* BC experts blind to the specific content of the BCIO will annotate intervention reports to identify all information they consider to be essential. The HBCP team will compare these annotations with the BCIO annotations to identify omissions or incompletely included information and discuss the results with the BC experts.
ii. *Inter-rater reliability of the manual annotation process.* The manual annotation will form the basis for training the automated annotator and so it is important that it be as accurate as possible. In the absence of an objective gold standard against which to assess accuracy, assessing inter-rater reliability will provide an index of likely accuracy. This can be achieved using methods similar to those already in place for identifying behaviour change techniques and modes of delivery [28, 29]. This involves calculating reliability statistics for sets of annotations.
iii. *Accuracy of the automated annotator.* Predictive accuracy of the automated annotator (i.e., its ability to match the study classifications of the manual annotations) will be assessed throughout the project through accuracy, precision and recall metrics, taking account of the hierarchical structure of the ontology and the inevitable dependency between classifications (e.g., a given outcome classification is highly likely to co-occur with a given intervention).
iv. *Accuracy of predictions from the ML and reasoning algorithms.* We will establish manually, by collaborating with behavioural change experts, a set of established effects and associated facts and will test the ML and reasoning algorithms against it by measuring the percentage of predictions that are in agreement.
v. *Comparison of BCI Knowledge System with existing methods of evidence synthesis.* We will create automated systematic reviews using the BCI Ontology to select relevant studies in conjunction with user input; use the automated data extraction and study evaluation tools to conduct syntheses and compare the results of this computer-assisted work with published systematic reviews, evaluating the automated reviews in terms of selection (are all the correct studies identified?), descriptive accuracy (are the studies correctly described and risk of bias correctly assessed?), and inferential claims (how do the conclusions compare with those from manually-conducted systematic reviews?)
vi. *User evaluation of the BCI Knowledge System’s accuracy, salience, validity, and utility.* Initially for domains with simple behaviours, robust outcome measures and relatively coherent evidence, we will use an International Organisation for Standardisation (ISO)-based evaluation framework [30] to evaluate the utility of the system as a whole. We will engage a range of decision-makers (e.g. practitioners, local government officers and national policymakers) and assess the extent to which the system is able to generate knowledge that addresses specific decisions.
vii. *New insights about behaviour change that are generated by the system.* We will assess the extent to which the system generates novel hypotheses and improved understanding of mechanisms of action.

## Discussion

The HBCP is an ambitious project aimed at developing and evaluating the first generation of a BCI Knowledge System. This will consist of a BCI Ontology, a set of processes and resources for manually annotating BCI evaluation reports according to this ontology to populate a BCI database, an automated annotator to achieve the annotation at scale with an acceptable level of accuracy for further populating the BCI database, a set of ML and reasoning algorithms to draw inferences from the BCI database, and an interface to allow users and other computer programs and to query and input to the knowledge base.

The first generation of the BCI Knowledge System will focus on synthesising and interpreting evidence from smoking cessation intervention evaluations in Cochrane reviews. The ontology will draw on established ontologies in related domains and be part of the OBO Foundry to maximise interoperability with other ontologies. An international network of stakeholders will be established to bring key experts and users into the development, evaluation and dissemination process. The BCI Knowledge System and its parts will undergo ongoing evaluation to inform its development and summative evaluation towards the end of the project to assess how far the project objectives have been met. It is hoped that the HBCP will represent the start of a new phase in behavioural and implementation science in which much more efficient use is made of the burgeoning research literature both for theory development and practical applications.

## Additional files

Additional file 1: Methodological approach to the development of the BCI Ontology, manual and automated annotation and machine learning and reasoning algorithms. (DOCX 30 kb)

Additional file 2: Methods for engaging stakeholders in the HBCP. (DOCX 23 kb)

## Abbreviations

AI: Artificial intelligence
API: Application programming interface
BCI: Behaviour change intervention
BCIO: Behaviour change intervention ontology
BCT: Behaviour change technique
HBCP: Human behaviour change project
HCI: Human-computer interface
ML: Machine Learning
NLP: Natural Language Processing
