# Supplementary material for: The Human Behaviour-Change Project: harnessing the power of artificial intelligence and machine learning for evidence synthesis and interpretation
Source: Implement Sci. 2017 Oct 18;12:121. doi: 10.1186/s13012-017-0641-5 (PMC5648456; doi:10.1186/s13012-017-0641-5)
Supplement: Supplementary file 1 — Methodological approach to the development of the BCI Ontology, manual and automated annotation and machine learning and reasoning algorithms. (DOCX 30 kb) [file 13012_2017_641_MOESM1_ESM.docx]

**Supplementary file 1: Methodological approach to the development of the BCI Ontology, manual and automated annotation and machine learning and reasoning algorithms**

*BCI Ontology development*

Development of the BCIO will involve extending existing taxonomies^g^ describing intervention content (including behaviour change techniques [1], and mechanisms of action [2] as well as adding new types of features to characterise intervention delivery (e.g., modes of delivery, dose), population (e.g., demographic characteristics), exposure (e.g., reach), setting (e.g., geographical region), behavioural targets (e.g., tobacco use), effects (e.g., size of effect), and study methods (e.g., risk of bias features). This will build on the current gathering of user feedback about the BCTTv1^g^ [1,3] via the web portal [4].

Semantic and putative causal relationships between entities in the BCIO will be defined (e.g., sub-class, positively influences).

The development of the BCIO will follow principles of the OBO Foundry [5] and align definitions of entities with the Basic Formal Ontology (BFO)^g^ [6]. BFO is a high-level ontology that contains universal terms, i.e. provides a structure that can be used across various domains. It has been used with success to develop domain-specific ontologies (e.g. the Gene Ontology [7] and the Mental Functioning Ontology [8]).

In developing the BCIO, we will aim to re-use entities and relationships from other established domain-specific ontologies as far as possible. The provenance (sources) of all parts of the BCIO will be recorded. We will register the BCIO with the OBO Foundry [5]. The BCI Ontology will be built using the Protégé ontology editor (currently at version 5.2) and stored and disseminated using the OWL^g^/XML^g^ format.

Table 1 shows examples of the top-level BCI Ontology entities identified in an evaluation of the effect on smoking cessation of adding a physical activity programme to standard behavioural support in pregnant smokers willing to try to stop smoking [9]. The labels are preliminary and designed to give an indication of the kind of entity involved. It is expected that each BCI evaluation report will involve several hundred instances of entities in the BCIO.

*Supplementary Table 1: Examples of BCIO entities identified in an evaluation of a physical activity intervention for smoking cessation [9]*

| **Top level BCIO entity label** | **BCIO entity label** | **Instance** |
| --- | --- | --- |
| Intervention | Intervention_content_specific | Supervised_treadmill-walking |
| Comparator | Comparator_content_specific | Advice_on_coping_with_craving |
| Population | Sample_intervention_age_mean | 27.2 |
| Setting | Setting_country | England |
| Exposure | Exposure_intervention_sessions_attended_median | 4 |
| Mechanisms of action | Mechanism_of_action_target | Craving_reduction |
| Target behaviour | Target_behaviour_type | Smoking_cessation |
| Outcome | Target_behaviour_intervention_% | 8 |
| Study method | Study_design | RCT_individual |
| Effect | Effect_size_type | Odds ratio |
| Study report | Study_author_first | Ussher, Michael |

The research will be iterative, identifying BCIO features in published BCI intervention evaluations, and identifying and adding to and revising the BCIO until a point is reached where it can be applied to new intervention evaluations without further modification.

In order to capitalise on studies at low risk of bias, we will initially limit evidence synthesis and interpretation to studies contained in reviews published by Cochrane^g^ [10]. Cochrane is in the process of encoding some of the features of their review studies using the PICO framework^g^ (Population/Intervention/Comparison/Outcome) with standard vocabulary terms to populate it [11]. We will work closely with Cochrane so that we can share annotations and methods.

*Manual annotation of BCI evaluation reports*

The process of developing the BCIO will require, and contribute to, the manual ‘annotation’ of BCI evaluation reports. Annotation involves identifying specific parts of text and/or tables and figures from a report and associating it with a ‘feature’ defined in terms of the BCIO. For example, the text in a report ‘The mean age of the intervention group was 25.3 years (SD 6.7)’ could be selected and associated with the BCIO entity whose label were given as ‘Intervention group mean age’, assigning it a value of ‘25.3’. Thus, a BCI evaluation report feature is the value assigned to a BCIO entity for that report. Two or more researchers will conduct annotations until a satisfactory level of inter-rater reliability is achieved.

The accumulating set of annotations will populate the BCI database^g^. Achieving a high level of accuracy of the manual annotations will be critical because errors will result in noise or bias when it comes to training the automated annotation system and when ML and reasoning algorithms are used to draw inferences from the BCI database.

Manual annotations will be undertaken by adding annotation functionality to our in-house software for systematic reviews, EPPI-Reviewer [12]. The fact that EPPI-Reviewer is used by thousands of systematic reviewers internationally means that it will become an important dissemination tool, enabling researchers in the BCI field to annotate their own reports according to the BCIO standard and contribute to the BCI database.

The BCI database will store information on the provenance of the annotations, saving not only the source but also a measure of confidence that the feature is correctly assigned. This will be important when it comes to training the automated annotation system and drawing inferences from the database.

Because manual annotation of reports is needed to develop the BCIO as well as to populate the BCI database, it is expected that early in the project annotations will go through multiple versions, with BCIO labels and definitions changing to accommodate new types of study in a way that maximises coherence across the full corpus of reports. At some point, however, it will be necessary to freeze existing entities in the BCIO and only change the BCIO by adding new entities within the existing structure.

*Development of automated annotation algorithms*

As the BCIO is being developed, and BCI reports are being manually annotated, we will begin to develop algorithms for automatically annotating BCI reports. These algorithms will use NLP tools and bespoke algorithms for reading text, tables and figures to work through BCI evaluation reports, identify BCIO entities and their values, and assign confidence ratings to each annotation. The algorithms will use manual annotations as training and testing sets, aiming to achieve the highest possible level of correspondence. The automated annotations and their provenance and confidence rating will be stored in the BCI database.

Because this part of the HBCP will proceed in parallel with the BCIO development and manual annotation of reports, we will begin training the automated annotator^g^ with manual annotations about which there is a high degree of confidence and where it is judged unlikely that the BCIO entities involved will change. Thus the BCIO, manual annotation and automated annotator will grow organically as more and more BCIO entities and associated annotations achieve a sufficient level of consistency.

Because BCI evaluation reports frequently have important information missing, or presented in a non-standard way, developing the automated annotator is likely to be the most challenging part of the HBCP, at least initially. However, we intend that the process of developing the BCIO and the automated annotator should provide an impetus to the field to become more consistent and comprehensive in its description of BCI evaluations. To that end, we will engage in extensive dissemination of the work as it progresses, and work closely with the HBCP Consortium to help create resources to assist researchers in presenting evaluation findings.

*Development of ML and reasoning algorithms*

While the BCI database generated by the automated annotator will itself be extremely useful for the field by allowing much more effective and efficient searching and querying of the BCI literature, the most novel advance offered by the HBCP is in the development of algorithms for evidence synthesis and interpretation. We will develop ML and reasoning algorithms that can respond to queries about intervention effectiveness by combining findings from individual studies at different levels of abstraction guided by the user, subject to the evidence being available.

For example, a user may ask ‘What is the effect on 6-month continuous smoking abstinence rates of brief opportunistic advice on smoking from a family practitioner given during a routine consultation to adult smokers in the UK?’. In this example, features specified to some degree and at some level of abstraction, are: type of intervention, delivery, target behaviour, population and setting. The query focuses on effect size as the BCIO entity that is unspecified. The reasoning algorithms will use the BCIO to search the BCI database for studies that match the specified features at the level of abstraction in the query, using semantic relationships such as ‘subclass of’ and ‘equivalent to’ in the ontology (e.g., family practitioner is equivalent to GP and subclass of physician). These algorithms will create a canonical form of the query together with a record of the inferences that were made to arrive at it. The latter is important because it affects the confidence rating in that the longer the inference chain, the lower the degree of confidence that the estimated feature values will apply to the features specified in the query.

In this example, algorithms will interrogate the database for features specified by the user, using the BCIO where needed to map study-specific features to features at the level of abstraction specified in the query, and generating predicted values for BCIO entities that are unspecified (e.g., the expected effect size) together with a rating of confidence in the estimate. The confidence estimate will not necessarily be the conventional ‘confidence interval’ or ‘credibility interval’ but may involve machine generated judgements of the probability (in a Bayesian sense) that the feature predicted is correct.

Having retrieved and organised data, ML algorithms may then need to predict the effect size from the canonical query and assign a confidence rating based on study method features (including risk of bias estimates) and inferences required to arrive at the canonical form of the query. The type of ML algorithm used should vary as a function of the data available to answer the query. In some cases, it may take the form of simple linear regression while in other cases much more complex non-parametric ML algorithms may be required.

Next, the ML and reasoning algorithms will need to be able to make use of available evidence in the BCI database while responding appropriately to situations where data are limited or non-existent. They will also need to respond appropriately to queries that are under-specified in the sense that at the level of abstraction of features in the query, a wide range of different answers are possible. Such a response could take the form of flagging significant heterogeneity in the BCI database, alerting the user to the fact that it is drawing on data from a more specific query, or guiding the user to be more specific in the query.

In the above example of GP smoking cessation advice, the response may be that the effect depends on the type of advice (e.g., offer of support with smoking cessation being more effective than just advising smokers to stop). The response may also flag up that most of the evidence comes from studies in smokers without a psychiatric diagnosis, or that the effect depends on the level of cigarette addiction in the target population.

At its heart, this process of developing the ML and reasoning algorithms will involve experimenting with several algorithms on a training set of reports and assessing how accurately they predict effects on a test set. Initial development, training and verification of the ML and reasoning algorithms will use a subset of reports in the Cochrane smoking cessation database, and a limited set of BCIO components. Once the algorithms have achieved an acceptable level of accuracy of prediction, we will gradually extend the training and testing sets into further subsets of smoking cessation intervention evaluations.

It will also be possible to assess the degree to which the automated ontology development improves the quality of the predictions. This process will be iterated to arrive at sets of algorithms that can be applied in an ongoing way to new reports and queries. At this point the learning will mostly take the form of updating parameters within a chosen set of algorithms unless and until accuracy of prediction becomes unacceptable, at which point it may be necessary to revisit the algorithms used.

It is expected that developing and training the ML and reasoning algorithms will require top-down input from behaviour change theory experts to constrain the search and estimation procedures. This will take the form both of focusing on features that are judged likely to be associated with each other and analysing cases where the ML and reasoning algorithms have failed to make accurate predictions.

Besides responding to queries about specific BCI scenarios it is intended that the ML and reasoning algorithms should generate new insights into behaviour change. This will be made possible by detecting patterns of association between values of entities within the BCIO. The process may occur as a background task, crawling through the database looking for patterns of association, or it may be guided by ‘theoretical queries’. In the case of brief opportunistic smoking cessation advice for example, the BCTs^g^ that form the content of that advice may be coded in many of the reports. ‘Goal setting’ may emerge as a commonly occurring BCT. Goal setting also occurs in other BCIs and so ML and reasoning algorithms could examine associations between goal setting and other BCI evaluation features to detect patterns indicating the circumstances in which goal setting is likely to be an important part of a BCI. This process may form part of an ongoing background task looking for patterns of associations involving BCTs, or it could be triggered by a query of the form ‘What role does goal setting play in behaviour change?’

Aside from making predictions and generating insights we intend that the ML and reasoning algorithms be able to report how they arrived at their conclusions, state important caveats and provide useful elaborations. This will be important for trust in the system, and for diagnostics to help determine why a prediction has failed in a particular scenario. To illustrate, in a response to the brief smoking cessation advice query, the conclusion that there is a high degree of confidence that ‘on average the effect size would be expected to be between a 1 and 3 percentage point increase’ could be supported by a statement of the kind ’Justification: A total of 25 high quality RCTs involving a total of 10,056 smokers were found that estimated the effect of brief smoking cessation advice by a physician on the specified outcome of which 4 were in the UK and 12 involved a family doctor. The effect size in the studies was not substantially affected by type of physician or country. Caveats: However, the mechanisms of action of the advice remain unclear and there is very little evidence in smokers diagnosed with a psychiatric disorder. Elaboration: A key part of the advice is setting a quit date within the next 4 weeks. The advice appears to be more effective if it includes offer of support in the form of a prescription for a stop-smoking medicine.’ Initially, the system would provide diagnostics to enable an expert to provide this interpretation. The development of a fully automated description would require further work on language synthesis and may be beyond the scope of the initial project, though we consider this to be attainable within a relatively short time horizon.

**References**

1. Michie S, Wood C, Johnston M, Abraham C, Francis J, Hardeman W. Behaviour Change Techniques: The Development and Evaluation of a Taxonomic Method for Reporting and Describing Behaviour Change Interventions. Health Technol Assess. 2015;19(99); 1-188.

2. Michie S, Carey RN, Johnston M, Rothman AJ, De Bruin M, Kelly MP. Connell L. From Theory-Inspired to Theory-Based Interventions: A Protocol for Developing and Testing a Methodology for Linking Behaviour Change Techniques to Theoretical Mechanisms of Action. Ann Behav Med. 2016: 1-12.

3. Michie S, Richardson M, Johnston M, Abraham C, Francis J, Hardeman W, Eccles MP, Cane J, Wood CE. The behavior change technique taxonomy (v1) of 93 hierarchically clustered techniques: building an international consensus for the reporting of behavior change interventions. Ann Beh Med. 2013;46:86-95.

4. Behaviour Change Techniques and Theory. [www.ucl.ac.uk/behaviour-change-techniques/bcttv1-feedback](http://www.ucl.ac.uk/behaviour-change-techniques/bcttv1-feedback). Accessed 21 July 2017.

5. OBO Foundry. [www.obofoundry.org](http://www.obofoundry.org). Accessed 21 July 2017.

6. Arp R, Smith B, Spear AD. Building ontologies with basic formal ontology. Cambridge, Massachusetts: MIT Press; 2015.

7. Ashburner M, Ball CA, Blake JA, Botstein D, Butler H, Cherry JM, Davis AP, Dolinski K, Dwight SS, Eppig JT, Harris MA, Hill DP, Issel-Tarver L, Kasarskis A, Lewis S, Matese JC, Richardson JE, Ringwald M, Rubin GM, Sherlock G. Gene Ontology: tool for the unification of biology. Nat Genet. 2000;25(1):25-9.

8. Hastings J, Smith B, Ceusters W, Jensen M, Mulligan K. Representing mental functioning: Ontologies for mental health and disease. ICBO 2012: 3rd International Conference on Biomedical Ontology; 2012: Citeseer.

9. Taylor A, West R, Ussher M, et al. The London Exercise And Pregnant smokers (LEAP) trial: a randomised controlled trial of physical activity for smoking cessation in pregnancy with an economic evaluation. 2015; 19(84): 1-135

10. Cochrane Collaboration. http://uk.cochrane.org. Accessed 21 July 2017.

11. Cochrane Collaboration. <http://linkeddata.cochrane.org/pico-ontology>. Accessed 21 July 2017.

12. Thomas J, Brunton J, Graziosi S. EPPI-Reviewer 4.0: software for research synthesis. London: Social Science Research Unit, Institute of Education; 2010.
