# Supplementary material for: The Human Behaviour-Change Project: harnessing the power of artificial intelligence and machine learning for evidence synthesis and interpretation
Source: Implement Sci. 2017 Oct 18;12:121. doi: 10.1186/s13012-017-0641-5 (PMC5648456; doi:10.1186/s13012-017-0641-5)
Supplement: Supplementary file 2 — Methods for engaging stakeholders in the HBCP. (DOCX 23 kb) [file 13012_2017_641_MOESM2_ESM.docx]

**Supplementary file 2: Methods for engaging stakeholders in the HBCP**

The HBCP views scientific, policy, provider and public global engagement as a vital part of its work and will engage a broad selection of interested parties using a range of methods and will provide an open platform to engage with the public globally. People will be guided to the website via creative use of a bespoke dissemination and publicity campaign (including the use of social media) to raise awareness of the HBCP. It is hoped that a wide range of people will become advocates of the project and contribute to global discussions on the behaviour change intervention ontology, annotation methods, artificial intelligence, machine learning and public engagement.

**Functions and Structure**

There are four functions that have been identified in previous discussions and preliminary papers:

1. High-level scientific, technical and strategic advice – Scientific Advisory Board
2. Behavioural Science expert feedback – Behavioural Science Expert Panel
3. Feedback on the project from a user perspective - Stakeholder User Group
4. Outreach and dissemination - Stakeholder Outreach Engagement Network
5. The **Scientific Advisory Board** will provide scientific, technical and strategic advice to the Human Behaviour-Change Project by:
   - Providing strategic and pragmatic advice
   - Acting as a sounding board
   - Pointing to relevant research programmes
   - Proposing areas of research or collaborations to pursue
   - Providing scrutiny of the research process and outputs
   - Help ensure engagement with and dissemination to relevant audiences and organisations

Because of the international nature of the Board, the majority of business will be conducted via webinar discussion and e-mail correspondence. Webinar/teleconference meetings will be called when required but are not expected to occur more than twice a year. Updates on the project will be provided and occasional feedback on plans and/or reports will be requested.

1. The **Behavioural Science Panel** will contribute scientific expertise to the developmental work of the HBCP on an ad hoc basis. It will inform the development of the Behaviour Change Intervention Ontology, by taking part in peer review and consensus exercises and will provide feedback about the functionalities and features of the HBCP user interface, from the perspective of behavioural scientists. The HBCP team will provide updates on the project on the study website or to participants directly.

The HBCP team will put materials online for peer review by the panel of experts; feedback will be in a variety of forms e.g. structured templates and online discussions. The estimated time for reviewing each material will be provided. Small group teleconferences/webinars may be held to discuss issues as they arise.

Panel members will respond on a voluntary basis and be expected to give feedback to the best of their ability. The amount of advisory input will depend on individual capacity. If the role proves to be too great a commitment, members can withdraw at any time. The contribution of the panel may be acknowledged on the study website, in disseminating findings from the project and in reporting to the Wellcome Trust; individuals can request for this not to happen.

1. The **Public Health Panel** will contribute scientific expertise to inform the development of the Population, Setting and Reach components of the Behaviour Change Intervention Ontology work. It will work in a similar way to the Behavioural Science Panel.
2. The **Stakeholder User Group**

Members will be purposively selected and invited to be part of a group of 20-30 people including policy makers, practitioners, researchers and members of the public. Members will be identified as potential users of one or more of the key outputs of the research e.g. the ontology, computer code, user interface. They will be enabled to follow the progress of the project over time and be asked to comment on aspects of its development from a user perspective. Activities could include:

- Providing information about relevant projects or potential collaborations
- Responding to online questions and polls
- End user testing
- Disseminating news updates and research findings to relevant audiences and organisations in order to increase the profile of the project and the research.

They will be engaged by occasional optional face-to-face meetings, focus groups or teleconference meetings (not more than twice a year). The HBCP team may occasionally request feedback on research plans and/or reports via email correspondence.

1. The **Stakeholder Outreach Engagement Network**

This network comprises everyone who signs up to the website (‘self-nominated’), either to receive the newsletter and/or to take part in a forum to comment on aspects of the project. There are no selection criteria, although materials will be mainly or all in the English language.

The purpose of the forum will be to release updates about the project’s progression, put issues out to vote, generate discussion in the websites open access forum and promote discussion. For those signing up to the forum, a special password-protected section within the website will be created.

Members will be able to:

- Drop comments in a virtual 'comments box'
- Respond to online polls and posted questions
